# Association of Body Mass Index and Body Mass Index Change with Mortality in Incident Peritoneal Dialysis Patients

**DOI:** 10.3390/nu7105405

**Published:** 2015-10-13

**Authors:** Liping Xiong, Shirong Cao, Fenghua Xu, Qian Zhou, Li Fan, Qingdong Xu, Xueqing Yu, Haiping Mao

**Affiliations:** 1 Department of Nephrology, The First Affiliated Hospital of Sun Yat-sen University, Key Laboratory of Nephrology, Ministry of Health of China, Guangdong Provincial Key Laboratory of Nephrology, Guangzhou 510080, China; xiongliping2000@126.com (L.X.); bcone@163.com (S.C.); lfan6262@gmail.com (L.F.); xqd82@163.com (Q.X.); yuxq@mail.sysu.edu.cn (X.Y.); 2 Epidemiology Research Unit and Translational Medicine Research Center, The First Affiliated Hospital of Sun Yat-sen University, Guangzhou 510080, China; xufenghua0411@126.com (F.X.); zhouqian2017@163.com (Q.Z.)

**Keywords:** continuous ambulatory peritoneal dialysis, body mass index, body mass index change, mortality

## Abstract

Although high body mass index (BMI) appears to confer a survival advantage in hemodialysis patients, the association of BMI with mortality in continuous ambulatory peritoneal dialysis (CAPD) patients is uncertain. We enrolled incident CAPD patients and BMI was categorized according to World Health Organization classification for Asian population. BMI at baseline and one year after the initiation of peritoneal dialysis (PD) treatment was assessed to calculate the BMI change (∆BMI). Patients were split into four categories according quartiles of ∆BMI. Kaplan-Meier method and Cox regression proportional hazard analysis were performed to assess the association of BMI on outcomes. A total of 1263 CAPD patients were included, with a mean age of 47.8 ± 15.0 years, a mean BMI of 21.58 ± 3.13 kg/m^2^. During a median follow-up of 25.3 months, obesity was associated with increased risk for cardiovascular diseases (CVD) death (adjusted hazard ratio (AHR) 2.01; 95% CI 1.14, 3.54), but not all-cause mortality. Additionally, patients with more BMI decline (>0.80%) during the first year after CAPD initiation had an elevated risk for both all-cause (AHR: 2.21, 95% CI 1.23–3.95) and CVD mortality (AHR 2.31, 95% CI 1.11, 4.84), which was independent of baseline BMI values.

## 1. Introduction

The World Health Organization (WHO) body mass index (BMI) categories of overweight and obesity are increasing globally. Many studies indicate that a high BMI is an independent risk factor for morbidity and mortality from cardiovascular disease (CVD) and certain cancers in the general population [1,2,3,4], and also elevates the risk for chronic kidney disease (CKD) as well as its progression to end-stage renal disease (ESRD) [5,6,7]. In contrast to the general population, an increased BMI is correlated with better outcomes in both prevalent and incident hemodialysis (HD) patients, including a lower risk of cardiovascular and all-cause mortality [8,9,10,11]. However, this “reverse epidemiology” or “paradoxical link” is controversial in peritoneal dialysis (PD) patients [12,13,14,15,16,17].

Most cohort studies of BMI and mortality published to date have been conducted in North American and European countries, few studies have been carried out in non-Western populations with the different BMI classification criteria. A large retrospective cohort study in the United States showed that PD patients with overweight or obese had lower or equivalent death rates relative to those with normal BMI [13]. The Australia and New Zealand Dialysis and Transplant (ANZDATA) analysis noted that obesity had adverse effects on both mortality and technical failure. Additionally, obesity in indigenous PD patients was independently associated with an increased risk for death and technique failure, whereas there was no significant relationship between BMI and mortality among patients of New Zealand Maori/Pacific Islander origin [14]. Moreover, high BMI in whites, African Americans, and Hispanics was found to be protective and associated with increased survival, even at extremely high BMI. Conversely, patients of Asians ethnic origin had worse mortality at high BMIs [18]. Accordingly, recent studies reported a U or J shaped curve in Chinese [19] and Korean [20] PD populations. In addition, data on BMI change in relation to mortality are limited in PD patients.

We conducted a retrospective cohort study to evaluate the association of BMI at the time of PD initiation and BMI change during the first year of PD therapy with mortality risk in Chinese CAPD patients.

## 2. Materials and Methods

### 2.1. Study Patients

We retrospectively reviewed the medical records of incident CAPD patients from our center between January 2006 and December 2011. Eligible patients were older than 18 years and had been on CAPD at least three months. We excluded patients who had a history of renal transplantation (*n* = 9), cancer (*n* = 12), or missing BMI data at baseline (*n* = 164). A total of 1263 patients were included in the final analysis. All patients were treated with glucose-based Dianeal solution (Baxter China Ltd., Guangzhou, China) and performed four exchanges per day of 2-L bags at the start of peritoneal dialysis.

### 2.2. Ethics Statement

This study was approved by the First Affiliated Hospital of Sun Yet-sen University Institutional Review Boards, and all subjects gave their written informed consent for participation.

### 2.3. Data Collection

All data used in this study were obtained from our database. Baseline demographic and clinical characteristics were collected at the initiation of PD therapy. Baseline BMI and laboratory parameters were evaluated three months after PD treatment. Presence of diabetes was defined as a self-reported history of physician diagnosis, the use of diabetes medication, or a fasting plasma glucose level of 126 mg/dL or greater in two separate occasions, or two-hour plasma glucose ≥200 mg/dL during the oral glucose tolerance [21]. Cardiovascular disease was defined as ischemic heart disease, congestive heart failure, cerebrovascular disease, and peripheral vascular disease, as previously described [22].

Body weight was assessed without PD fluid in the abdominal cavity. BMI was calculated as weight in kilograms divided by the square of height in meters (kg/m^2^) and categorized according to the World Health Organization (WHO) classification for Asian population: underweight (<18.5 kg/m^2^), normal weight (18.5–22.9 kg/m^2^), overweight (23.0–24.9 kg/m^2^), and obese (≥25.0 kg/m^2^). To investigate the association of BMI change (ΔBMI) and mortality, BMI at 3 (BMI_1_) and at 15 months (BMI_2_) were defined as baseline BMI and one year after the initiation of PD treatment, respectively. The calculation of BMI change was based on following formula: ΔBMI = (BMI_2_ − BMI_1_)/BMI_1_. To this end, 153 patients with missing data on BMI at last survey were excluded. We excluded an additional 230 patients who were treated with PD for less than one year due to death (*n* = 73), transfer to HD (*n* = 26), and renal transplantation (*n* = 85). We also excluded patients who transferred to other centers (*n* = 26) or were lost to follow-up (*n* = 20). Thus, 880 of 1263 patients were included in this sub-analysis. The patients were split into four groups according quartiles (Q) of ΔBMI: Q1 (<−0.80%); Q2 (−0.80%–2.69%, reference category); Q3 (2.70%–7.40%); Q4 (>7.40%).

Measurements of both peritoneal and renal Kt/V urea and creatinine clearances and residual glomerular filtration rate (rGFR) were calculated by using PD Adequest 2.0 for Windows software (Baxter Healthcare Corporation, Chicago, IL, USA). All biochemical and hematological tests were measured in the biochemical laboratory of the First Affiliated Hospital of Sun Yat-sen University.

### 2.4. Outcomes

The primary outcomes were all-cause and cardiovascular mortality. Cardiovascular mortality was defined as death from congestive heart failure, ischemic heart disease, cerebrovascular disease, peripheral vascular disease, arrhythmia (all of which were defined according to standard clinical criteria), and sudden death [23,24,25]. Sudden death was diagnosed as unexpected natural death occurring within one hour of the onset of symptoms or without any prior condition that appear fatal [22]. Survival time was defined as the time from enrollment to death or administrative censoring including transfer to hemodialysis, renal transplantation, transfer to other dialysis centers, loss to follow-up, or end of the study period at 31 December 2012.

### 2.5. Statistical Analyses

Continuous variables are expressed as mean ± SD or median (interquartile range), and categorical variables are expressed as number and percentages. Differences in demographic and clinical characteristics among baseline BMI categories were evaluated using analysis of variance (ANOVA), Kruskal-Wallis test or chi-square test appropriately.

Survival rates were calculated by patient years. The survival curves were drawn by Kaplan-Meier method and the difference between distributions of survival of baseline BMI categories or BMI change groups were assessed by Log-rank test. Cox proportional hazards models were used to estimate the associations of baseline BMI or BMI change with all-cause and cardiovascular mortality, and to obtain the hazard ratios (HR), adjusted hazard ratios (AHRs), and their 95% confidence intervals (95% CI). The multivariable Cox regression models were conducted using covariates that significant association with mortality or for importance of clinical concern including age, sex, diabetes, history of CVD, mean arterial pressure (MAP), hemoglobin, serum albumin, total cholesterol (TC), triglyceride (TG), high sensitive C-reactive protein (hs-CRP), rGFR and Kt/V_urea_ (urea clearance (Kt) normalized to total body water (V)). All statistical analyses were performed using SPSS software for Windows, version 13.0 (SPSS Inc., Chicago, IL, USA). A *p*-value < 0.05 was considered statistically significant.

## 3. Results

### 3.1. Patient Characteristics at Baseline

A total of 1263 incident CAPD patients were enrolled, with a mean age of 47.8 ± 15.0 years, a mean BMI of 21.58 ± 3.13 kg/m^2^; 24.1% were diabetes and 48.2% had history of CVD at baseline. According to the WHO classification for Asia population, 15.1% were underweight, 56.0% were normal weight, 16.0% were overweight, and 12.9% were obese. Table 1 represented the baseline demographic and clinical data of the studied cohort by BMI category. In the entire cohort, overweight and obese subjects were more likely to be older and had higher proportion of diabetes, as compared with normal weight individuals. There was a trend towards higher levels of TG, hs-CRP, and rGFR with increased BMI values (all *p* < 0.001), whereas BMI categories were inversely related to total Kt/V_urea_ (*p* < 0.001). There were no significant differences among groups with respect to CVD history, MAP, hemoglobin, serum albumin, and TC.

**Table 1:** Baseline characteristics of the study population by BMI categories.

Variable *	Underweight	Normal Weight	Overweight	Obesity	*p* Value
Number of patients	191 (15.1)	707 (56.0)	202 (16.0)	163 (12.9)	
BMI (kg/m^2^)	17.27 ± 0.99	20.74 ± 1.20	23.94 ± 0.58	27.31 ± 1.92	<0.001
Age (years)	43.52 ± 16.23	46.82 ± 14.94	52.43 ± 13.81	51.01 ± 13.13	<0.001
Male	71 (37.2)	436 (61.7)	129 (63.9)	104 (63.8)	<0.001
Cause of ESRD					<0.001
Glomerulonephritis	148 (77.5)	457 (64.6)	91 (45.0)	74 (45.4)	
Diabetic nephropathy	16 (8.4)	137 (19.4)	68 (33.7)	55 (33.7)	
Hypertension	6 (3.1)	49 (6.9)	23 (11.4)	20 (12.3)	
Other	21 (11.0)	64 (9.1)	20 (9.9)	14 (8.6)	
Diabetes	18 (9.4)	150 (21.2)	77 (38.1)	60 (36.8)	<0.001
History of CVD	84 (44)	348 (49.2)	97 (48.0)	80 (49.1)	0.634
MAP (mmHg)	101.3 ± 13.6	102.4 ± 14.2	103.1 ± 13.3	104.0 ± 13.5	0.299
Hemoglobin (g/L)	110.8 ± 23.4	108.6 ± 22.8	109.1 ± 21.6	104.0 ± 13.9	0.086
Albumin (g/L)	37.91 ± 5.66	37.89 ± 4.93	38.22 ± 4.87	37.87 ± 5.34	0.866
TC (mmol/L)	5.19 (4.40–5.94)	5.00 (4.30–5.80)	4.95 (4.20–5.98)	5.20 (4.39–6.30)	0.076
TG (mmol/L)	1.24 (0.94–1.75)	1.36 (0.94–1.82)	1.63 (1.53–2.35)	1.98 (1.46–3.14)	<0.001
hs-CRP (mg/L)	0.86 (0.38–4.88)	1.45 (0.55–4.40)	3.12 (1.45–8.99)	3.16 (1.40–10.39)	<0.001
rGFR (mL/min/1.73m^2^)	2.54 (1.20–3.60)	2.83 (1.51–4.48)	3.45 (2.06–5.28)	3.26 (1.74–5.14)	<0.001
Kt/V_urea_	2.50 ± 0.57	2.36 ± 0.63	2.34 ± 0.64	2.20 ± 0.61	0.001

* Variables are presented as mean ± SD, median (interquartile range), or number (proportion). Abbreviation: BMI, body mass index; ESRD, end stage renal disease; CVD, cardiovascular disease; MAP, mean arterial pressure; TC, total cholesterol; TG, triglyceride; hs-CRP, high sensitive C-reactive protein; rGFR, residual glomerular filtration rate; Kt/V_urea_, urea clearance (Kt) normalized to total body water (V).

### 3.2. BMI and Patient Survival

**Figure 1:**
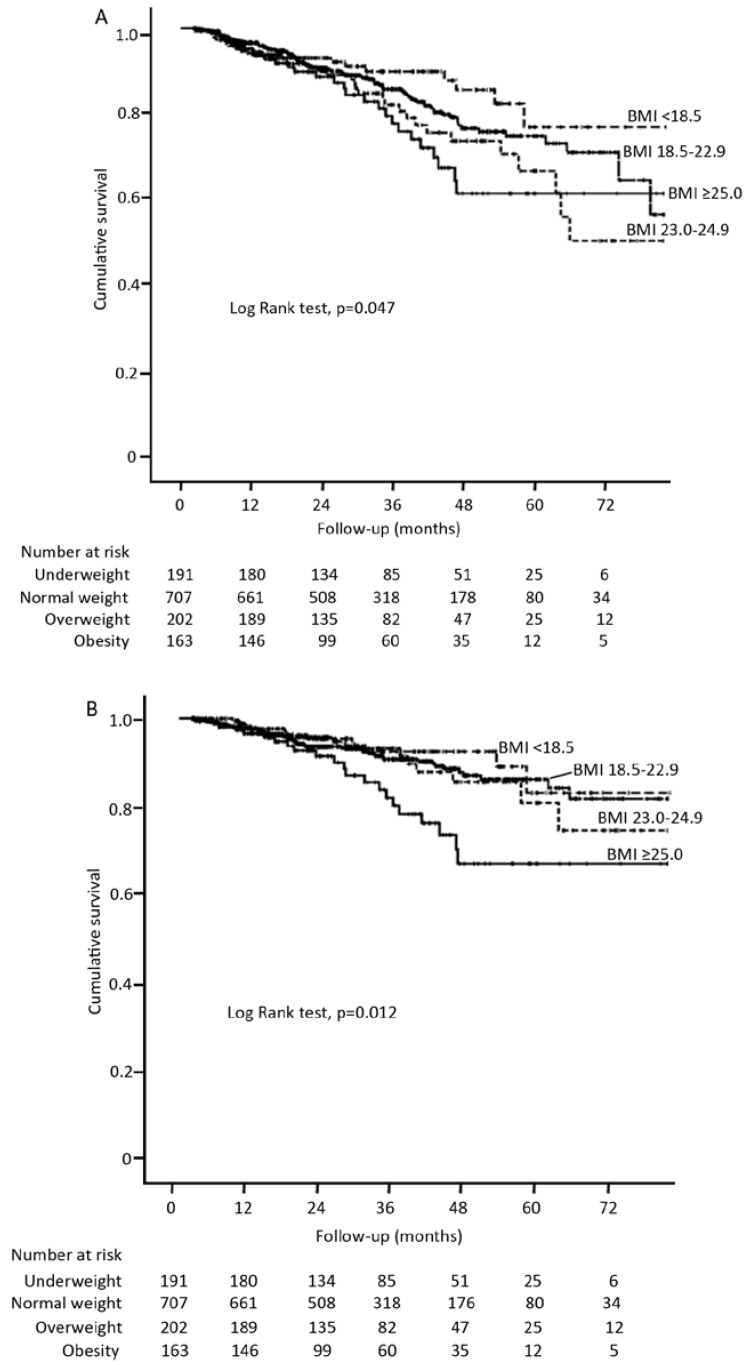
Kaplan Meier curves for all-cause mortality (**A**) and cardiovascular mortality (**B**) in incident continuous ambulatory peritoneal dialysis (CAPD) patients by BMI categories.

During a median follow-up of 25.3 months (range 3.03–82.07), 176 patients (13.9%) died. Of them, 59.1% were attributable to cardiovascular causes, 6.3% were due to PD-related peritonitis, 34.6% to others (including no-PD-related infection, malignancies, cachexia, gastrointestinal bleeding, withdrawal from dialysis). Kaplan-Meier survival curve analysis showed significant lower overall survival (Figure 1A) and cardiovascular death-free survival (Figure 1B) in the obese patients, as compared with the other three BMI categories. Likewise, both all-cause and cardiovascular mortality rates increased in each successively higher BMI category, with lowest mortality rates in underweight patients (4.0 and 2.5 death/100 patient-years (pys), respectively) and highest in that of obese subjects (8.0 and 6.3 death/100 pys, respectively). Obesity was unadjusted associated with increased risk for all-cause and cardiovascular mortality (HR, 1.57 (1.03–2.39) and 2.07 (1.26–3.40), respectively), compared with patients with normal weight. However, these associations persisted for CVD death (HR, 2.01 (1.14–3.54), *p* = 0.004) but not for all-cause mortality after adjustment for covariates (Table 2), suggesting a negative effect of obesity in CVD mortality among PD population. The results were similar when BMI was modeled as continuous variable in Cox regression hazards model. The risk of cardiovascular mortality increased steadily by 9% for every unit increase in BMI (HR, 1.09 (1.01–1.18), *p* = 0.036).

**Table 2:** Association between baseline BMI categories and all-cause and cardiovascular mortality.

Variable	Univariate		Multivariate *	
	HR (95% CI)	*p* Value	HR (95% CI)	*p* Value
All-cause mortality				
Underweight	0.75 (0.46–1.22)	0.246	0.61 (0.30–1.25)	0.178
Normal weight	1.00 (ref.)		1.00 (ref.)	
Overweight	1.33 (0.90–1.97)	0.153	0.99 (0.61–1.59)	0.949
Obesity	1.57 (1.03–2.39)	0.038	1.54 (0.94–2.52)	0.085
Cardiovascular mortality				
Underweight	0.81 (0.44–1.52)	0.516	0.79 (0.35–1.81)	0.582
Normal weight	1.00 (ref.)		1.00 (ref.)	
Overweight	1.03 (0.57–1.79)	0.973	0.78 (0.41–1.49)	0.437
Obesity	2.07 (1.26–3.40)	0.004	2.01 (1.14–3.54)	0.016

Abbreviations and definitions as listed in Table 1. HR: hazard ratio. * adjusted for age, gender, diabetes, CVD, MAP, hemoglobin, albumin, TC, TG, Hs-CRP, rGFR, Kt/V_urea_.

### 3.3. BMI Change and Mortality

**Figure 2:**
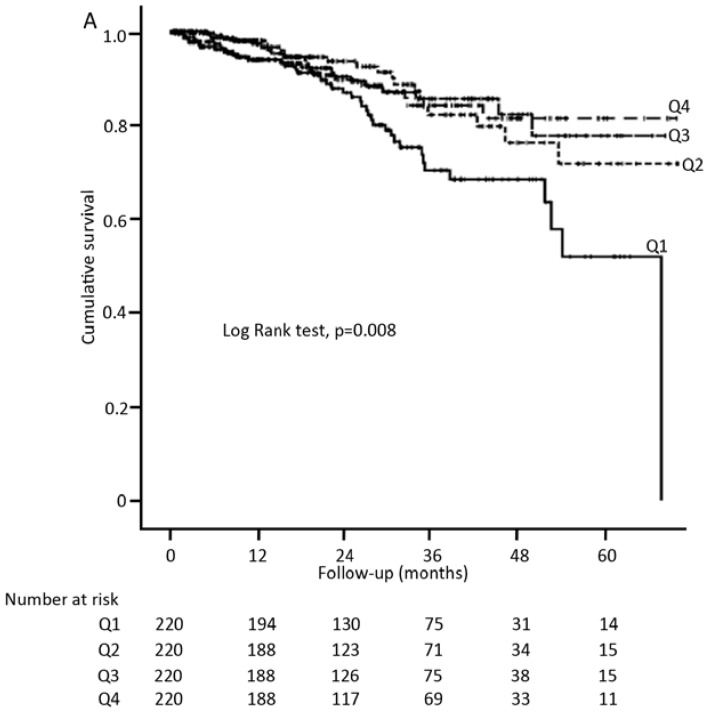

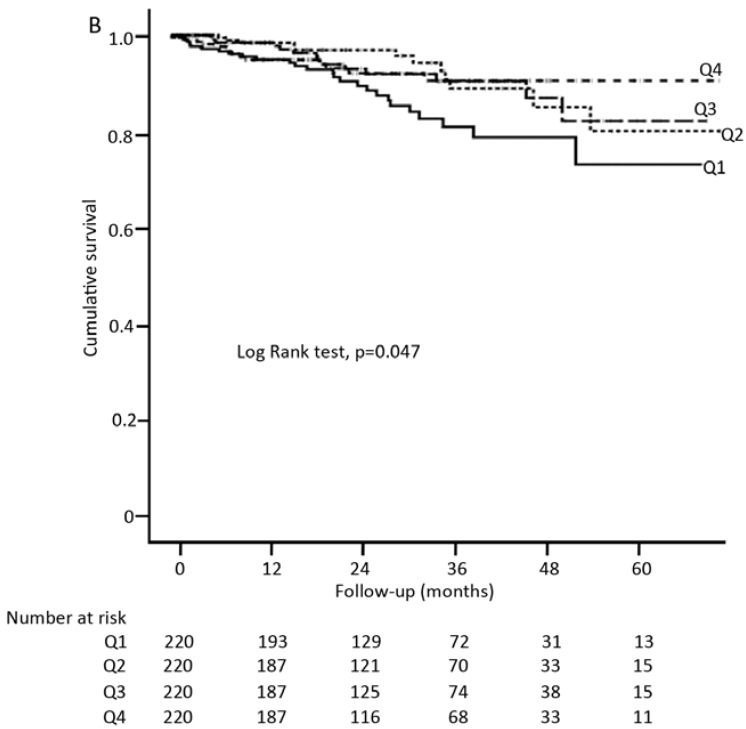
Kaplan Meier curves for all-cause mortality (**A**) and cardiovascular mortality (**B**) in the subgroup of 880 patients by BMI change categories.

Table 3 showed the baseline characteristics of patients stratified by the quartiles distribution of ΔBMI (Q1, Q2, Q3 and Q4). There were no significant differences in baseline BMI categories and other demographic or clinical data across quartiles of ΔBMI, except for baseline BMI values, in which patients in the highest quartile of ΔBMI (Q4) tended to have lower baseline BMI, suggesting that patients in Q4 group had an increased BMI levels after the first year of PD treatment. Notably, patients in the lowest quartile of ΔBMI (Q1) were likely to reduce absolute or relative BMI values. Figure 2 depicted the survival curves for all-cause (A) and cardiovascular mortality (B) in the subgroup of 880 patients across quartiles of ΔBMI, revealing that patients in Q1 group had the worst prognosis, compared to those with relatively stable BMI levels (Q2). Table 4 showed the Cox proportional hazard for the BMI change, with the Q2 group as a reference. Consistently, only patients in Q1 group were at increased risk of both all-cause (HR 2.01 (1.18–3.41)) and cardiovascular mortality (HR 2.24 (1.13–4.44)), whereas no impact was observed among other groups. This negative association was even more prominent after adjusting for the covariates.

**Table 3:** Baseline characteristics of the study population by BMI change categories.

Variable *	Q1 (<−0.80%)	Q2 (−0.80%–2.69%)	Q3 (2.70%–7.40%)	Q4 (>7.40%)	*p* Value
Patients (*n*)	220	220	220	220	
Baseline BMI (kg/m^2^)	21.73 ± 3.1	21.87 ± 2.99	21.56 ± 3.01	20.87 ± 2.98	0.003
Baseline BMI group					0.084
Underweight	32 (14.6)	27 (12.3)	31 (14.1)	46 (20.9)	
Normal weight	121 (55.0)	126 (57.3)	125 (56.8)	132 (60.0)	
Overweight	36 (16.4)	38 (17.3)	40 (18.2)	22 (10.0)	
Obesity	31 (14.1)	29 (13.2)	24 (10.9)	20 (9.1)	
Age (years)	48.1 ± 15.8	48.1 ± 14.7	46.9 ± 14.3	48.3 ± 14.5	0.757
Male	122 (55.5)	129 (58.6)	139 (63.2)	111 (50.5)	0.052
Diabetes	55 (25.0)	56 (25.5)	41 (18.6)	56 (25.5)	0.253
History of CVD	102 (46.4)	106 (48.2)	100 (45.5)	116 (52.7)	0.429
MAP (mmHg)	103.1 ± 14.2	101.4 ± 13.2	101.9 ± 13.0	102.5 ± 12.5	0.519
Hemoglobin (g/L)	111.1 ± 21.1	110.1 ± 22.2	112.1 ± 21.5	110.5 ± 21.8	0.791
Albumin (g/L)	38.2 ± 4.7	38.3 ± 4.7	39.0 ± 5.0	38.2 ± 5.1	0.244
TC (mmol/L)	5.20 (4.40–5.90)	5.00 (4.30–5.98)	5.07 (4.30–5.88)	5.20 (4.30–6.20)	0.785
TG (mmol/L)	1.54 (1.06–2.23)	1.44 (1.05–2.03)	1.36 (0.95–2.05)	1.42 (1.05–1.98)	0.372
hs-CRP (mg/L)	1.73 (0.58–6.34)	1.90 (0.58–6.14)	1.96 (0.53–5.20)	1.78 (0.72–5.51)	0.591
rGFR (mL/min/1.73m^2^)	3.08 (1.47–5.04)	3.04 (1.65–4.40)	2.92 (1.66–4.49)	2.95 (1.21–4.75)	0.704
Kt/V_urea_	2.40 ± 0.67	2.33 ± 0.66	2.35 ± 0.65	2.38 ± 0.56	0.654

* Variables are presented as mean ± SD, median (interquartile range), or number (proportion). Abbreviation and definitions as listed in Table 1.

**Table 4:** Association between BMI change categories and all-cause and cardiovascular mortality.

Variable	Univariate		Multivariate *	
	HR (95% CI)	*p* Value	HR (95% CI)	*p* Value
All-cause mortality				
Q1	2.01 (1.18–3.41)	0.010	2.21 (1.23–3.95)	0.008
Q2	1.00 (ref.)		1.00 (ref.)	
Q3	0.96 (0.52–1.78)	0.903	1.09 (0.54–2.19)	0.818
Q4	1.10 (0.61–2.01)	0.747	1.19 (0.63–2.26)	0.600
Cardiovascular mortality				
Q1	2.24 (1.13–4.44)	0.021	2.31 (1.11–4.84)	0.026
Q2	1.00 (ref.)		1.00 (ref.)	
Q3	1.17 (0.54–2.53)	0.693	1.23 (0.58–3.23)	0.479
Q4	1.21 (0.56–2.61)	0.630	1.22 (0.53–2.77)	0.643

HR: hazard ratio. * adjusted for age, gender, diabetes, CVD, baseline BMI, MAP, hemoglobin, albumin, TC, TG, HSCRP, rGFR, Kt/V_urea_.

## 4. Discussion

This study investigated the association of mortality with baseline BMI and BMI change in a cohort of incident peritoneal dialysis patients with BMI categories according to the WHO classification for Asian population. Our findings suggest that being obese was independently associated with increased risk of CVD death, but not all-cause mortality compared to normal BMI status. Each unit elevation in BMI conferred a 9% higher risk of cardiovascular mortality. Notably, during the first year after CAPD initiation, patients with more BMI decline (>0.80%) had an increased risk for all-cause and CVD mortality, which was independent of baseline BMI values. These findings remained virtually significant after adjusting for confounders.

A body of evidence has demonstrated that a higher BMI is more susceptible to cardiovascular and other chronic disease in general population. In CKD patients, obesity is also recognized as a risk factor for the progression to ESRD [5,6,7]. However, an inverse association between obesity and mortality has been documented in HD patients. Nevertheless, the influence of BMI on survival in PD patients is uncertain. These conflicting results might be due to the difference in the BMI classification criteria, a wide variety of BMI categories and varying reference categories, racial origin, a mixture of HD and PD patients, and comorbid illness burden.

Drawing from the United States Renal Data System, Abbott *et al.* [26] and Stack *et al.* [27] reported that the association between high BMI and survival in chronic dialysis patients differed by dialysis modality. In adjusted analysis, BMI ≥ 30 kg/m^2^ was associated with improved survival in HD patients but not PD patients [26,27]. Another large study, which included 418,055 patients from different race and ethnicities, showed that high BMI was associated with increased survival for white, African Americans, and Hispanics. Nevertheless, opposite results were found in Asian PD patients [18], indicating that race may affects outcomes in PD patients with high BMI. In contrast, several studies did not found any relation between high BMI and all-cause mortality in Western populations [16,17,28]. Additionally, using WHO BMI classification for Asian populations, a recent study included 274 Chinese PD population revealed a U-shaped relationship between BMI and mortality, with higher mortality in the underweight and obese patients [19]. However, in a study of 900 Koreans, an increased risk death was observed in BMI values lower than 21.4, whereas no association was found in the higher BMI (>23.5 kg/m^2^) [20]. Consistent with this, the adjustment for potential confounding factors attenuated and eliminated the relationship between obesity and all-cause mortality in our study. We were also unable to show an association between underweight or overweight and all-cause death.

Cardiovascular disease is the leading cause of death in patients with ESRD, and obesity has been implicated as a major risk factor for cardiovascular health [29,30,31,32,33]. In the present study, the highest risk death from CVD was found in obese PD patients, yet underweight patients was not associated with increased mortality risk. As discussed above, our data showed that obesity was not a significant predictor for all-cause mortality. This may be largely explained by our observation that the underlying cause for the majority (59.1%) of death was attributable to cardiovascular disease. Additionally, obesity has been strongly associated with other cardiovascular risk factors, such as hypertension, diabetes, hypercholesterolemia, and pro-inflammatory state [34,35,36,37]. Indeed, we found that patients with obesity had a higher proportion of diabetes, greater levels of total cholesterol, triglyceride, and hs-CRP compared to those with normal weight. Thus, it may be partly accounted for the elevated risk of cardiovascular mortality in obese PD patients to some extent, although we took these potential confounding factors into account in analyses.

Few studies have explored the relation between BMI change and mortality. We observed BMI decrease >0.80% during the first year of PD therapy was associated with significantly elevated both all-cause and CVD mortality regardless of baseline BMI, when compared to those with a relative stable BMI values. The results suggested that a reduction of BMI more than 0.80% was an independent predictor of poor prognosis among patients who were in any of the BMI categories at baseline. Nevertheless, BMI reducing less than 0.80% and BMI increasing had no excess mortality risk association. Similarly, the study by Kalantar-Zadeh *et al.* in a cohort of 54,535 HD patients from virtually all DaVita dialysis clinics in the United States found that during a two-year follow-up, the risk of death was significantly increased among patients with weight loss more than 1% per annual quarter, but there was no association of weight gain with mortality [38]. Fernandes *et al.* also reported that a body weight loss >3.1% during the first year of PD treatment was associated with significantly increased mortality [17]. Weight loss, one of the most characteristic signs of malnutrition, is a common finding in patients with chronic disease and associated with poor prognosis. In our study, although the levels of albumin were not significantly different across BMI change categories, whether other markers of nutrition had a BMI change related difference and their effects on mortality has been unclear.

One of the strengths of our study is the large sample size to evaluate association of baseline BMI and BMI change over time with mortality in Chinese PD populations. However, our study had limitations. First, we used BMI data, not weight data. BMI does not distinguish weight from muscle or fat. We only addressed BMI-related mortality. Thus, our results may not necessarily extend to relation between weight and mortality. Second, this study did not adjust for unintentional weight loss. Third, a median follow-up duration of 25 months was relatively short to determine the association of BMI and all-cause and CVD mortality, and accuracy of the data on the death certification might be uncertain. It was unclear whether the impact of BMI reduction on mortality persisted over time. Fourth, because waist to hip ratio, visceral fat measurement, and proportion of body fat were unavailable, we could not exclude the potential prognostic role of these other important indices of adiposity. Fifth, smoking may affect subjects’ body weight and itself a strong independent predictor of all-cause and cardiovascular mortality. The detailed data regarding patients’ smoking history and amount were unavailable; hence, our study cannot exclude this critical confounding influence of smoking on the association of BMI change with death risk. Finally, a retrospective observational study cannot exclude the possibility of unmeasured confounding.

## 5. Conclusions

In conclusion, obesity at baseline is associated with higher cardiovascular mortality in Chinese CAPD patients. Further, BMI decrease more than 0.80% during the first year of PD therapy may confer an independent risk for both all-cause and CVD death in this population. These findings suggest that strategies to reduce mortality need to be targeted not only to obese PD patients but also toward those with a greater decrease in BMI.

## References

[1] Berrington de Gonzalez A., Hartge P., Cerhan J.R., Flint A.J., Hannan L., MacInnis R.J., Moore S.C., Tobias G.S., Anton-Culver H., Freeman L.B. (2010). Body-mass index and mortality among 1.46 million white adults. N. Engl. J. Med..

[2] Calle E.E., Thun M.J., Petrelli J.M., Rodriguez C., Heath C.W. (1999). Body-mass index and mortality in a prospective cohort of U.S. adults. N. Engl. J. Med..

[3] Stevens J., Cai J., Pamuk E.R., Williamson D.F., Thun M.J., Wood J.L. (1998). The effect of age on the association between body-mass index and mortality. N. Engl. J. Med..

[4] Byers T. (1995). Body weight and mortality. N. Engl. J. Med..

[5] Hsu C.Y., McCulloch C.E., Iribarren C., Darbinian J., Go A.S. (2006). Body mass index and risk for end-stage renal disease. Ann. Intern. Med..

[6] Nguyen S., Hsu C.Y. (2007). Excess weight as a risk factor for kidney failure. Curr. Opin. Nephrol. Hypertens..

[7] Praga M., Morales E. (2006). Obesity, proteinuria and progression of renal failure. Curr. Opin. Nephrol. Hypertens..

[8] Chazot C., Gassia J.P., di Benedetto A., Cesare S., Ponce P., Marcelli D. (2009). Is there any survival advantage of obesity in southern European haemodialysis patients?. Nephrol. Dial. Transplant..

[9] Kalantar-Zadeh K., Abbott K.C., Salahudeen A.K., Kilpatrick R.D., Horwich T.B. (2005). Survival advantages of obesity in dialysis patients. Am. J. Clin. Nutr..

[10] Port F.K., Ashby V.B., Dhingra R.K., Roys E.C., Wolfe R.A. (2002). Dialysis dose and body mass index are strongly associated with survival in hemodialysis patients. J. Am. Soc. Nephrol..

[11] Schmidt D.S., Salahudeen A.K. (2007). Obesity—Survival paradox—Still a controversy?. Semin. Dial..

[12] Johnson D.W., Herzig K.A., Purdie D.M., Chang W., Brown A.M., Rigby R.J., Campbell S.B., Nicol D.L., Hawley C.M. (2000). Is obesity a favorable prognostic factor in peritoneal dialysis patients?. Perit. Dial. Int..

[13] Snyder J.J., Foley R.N., Gilbertson D.T., Vonesh E.F., Collins A.J. (2003). Body size and outcomes on peritoneal dialysis in the United States. Kidney Int..

[14] McDonald S.P., Collins J.F., Johnson D.W. (2003). Obesity is associated with worse peritoneal dialysis outcomes in the Australia and New Zealand patient populations. J. Am. Soc. Nephrol..

[15] Unal A., Sipahioglu M.H., Kocyigit I., Elmali F., Tokgoz B., Oymak O. (2014). Does body mass index affect survival and technique failure in patients undergoing peritoneal dialysis?. Pak. J. Med. Sci..

[16] De Mutsert R., Grootendorst D.C., Boeschoten E.W., Dekker F.W., Krediet R.T. (2009). Is obesity associated with a survival advantage in patients starting peritoneal dialysis?. Contrib. Nephrol..

[17] Fernandes N.M., Bastos M.G., Franco M.R., Chaoubah A., Lima Mda G., Divino-Filho J.C., Qureshi A.R. (2013). Body size and longitudinal body weight changes do not increase mortality in incident peritoneal dialysis patients of the Brazilian peritoneal dialysis multicenter study. Clinics.

[18] Johansen K.L., Young B., Kaysen G.A., Chertow G.M. (2004). Association of body size with outcomes among patients beginning dialysis. Am. J. Clin. Nutr..

[19] Kiran V.R., Zhu T.Y., Yip T., Lui S.L., Lo W.K. (2014). Body mass index and mortality risk in Asian peritoneal dialysis patients in Hong Kong—Impact of diabetes and cardiovascular disease status. Perit. Dial. Int..

[20] Kim Y.K., Kim S.H., Kim H.W., Kim Y.O., Jin D.C., Song H.C., Choi E.J., Kim Y.L., Kim Y.S., Kang S.W. (2014). The association between body mass index and mortality on peritoneal dialysis: A prospective cohort study. Perit. Dial. Int..

[21] Alberti K.G., Zimmet P.Z. (1998). Definition, diagnosis and classification of diabetes mellitus and its complications. Part 1: Diagnosis and classification of diabetes mellitus provisional report of a who consultation. Diabet. Med..

[22] Xu Q., Xiong L., Fan L., Xu F., Yang Y., Li H., Peng X., Cao S., Zheng Z., Yang X. (2014). Association of pulmonary hypertension with mortality in incident peritoneal dialysis patients. Perit. Dial. Int..

[23] Psaty B.M., Kuller L.H., Bild D., Burke G.L., Kittner S.J., Mittelmark M., Price T.R., Rautaharju P.M., Robbins J. (1995). Methods of assessing prevalent cardiovascular disease in the cardiovascular health study. Ann. Epidemiol..

[24] Shlipak M.G., Sarnak M.J., Katz R., Fried L.F., Seliger S.L., Newman A.B., Siscovick D.S., Stehman-Breen C. (2005). Cystatin C and the risk of death and cardiovascular events among elderly persons. N. Engl. J. Med..

[25] Wang A.Y., Wang M., Woo J., Lam C.W., Li P.K., Lui S.F., Sanderson J.E. (2003). Cardiac valve calcification as an important predictor for all-cause mortality and cardiovascular mortality in long-term peritoneal dialysis patients: A prospective study. J. Am. Soc. Nephrol..

[26] Abbott K.C., Glanton C.W., Trespalacios F.C., Oliver D.K., Ortiz M.I., Agodoa L.Y., Cruess D.F., Kimmel P.L. (2004). Body mass index, dialysis modality, and survival: Analysis of the United States renal data system dialysis morbidity and mortality wave II study. Kidney Int..

[27] Stack A.G., Murthy B.V., Molony D.A. (2004). Survival differences between peritoneal dialysis and hemodialysis among “large” ESRD patients in the United States. Kidney Int..

[28] Fried L., Bernardini J., Piraino B. (1996). Neither size nor weight predicts survival in peritoneal dialysis patients. Perit. Dial. Int..

[29] Chen Y., Copeland W.K., Vedanthan R., Grant E., Lee J.E., Gu D., Gupta P.C., Ramadas K., Inoue M., Tsugane S. (2013). Association between body mass index and cardiovascular disease mortality in east Asians and south Asians: Pooled analysis of prospective data from the Asia cohort consortium. BMJ.

[30] Chen Z., Yang G., Offer A., Zhou M., Smith M., Peto R., Ge H., Yang L., Whitlock G. (2012). Body mass index and mortality in China: A 15-year prospective study of 220,000 men. Int. J. Epidemiol..

[31] Jee S.H., Sull J.W., Park J., Lee S.Y., Ohrr H., Guallar E., Samet J.M. (2006). Body-mass index and mortality in Korean men and women. N. Engl. J. Med..

[32] Billington C.J., Epstein L.H., Goodwin N.J., Hill J.O., Pi-Sunyer F.X., Rolls B.J., Stern J., Wadden T.A., Weinsier R.L., Wilson G.T. (2000). Overweight, obesity, and health risk. Arc. Intern. Med..

[33] Asia Pacific Cohort Studies Collaboration (2004). Body mass index and cardiovascular disease in the Asia-Pacific region: An overview of 33 cohorts involving 310,000 participants. Int. J. Epidemiol..

[34] Memish Z.A., el Bcheraoui C., Tuffaha M., Robinson M., Daoud F., Jaber S., Mikhitarian S., Al Saeedi M., AlMazroa M.A., Mokdad A.H. (2014). Obesity and associated factors—Kingdom of Saudi Arabia, 2013. Prev. Chronic Dis..

[35] Haslam D.W., James W.P. (2005). Obesity. Lancet.

[36] Nikolic D., Katsiki N., Montalto G., Isenovic E.R., Mikhailidis D.P., Rizzo M. (2013). Lipoprotein subfractions in metabolic syndrome and obesity: Clinical significance and therapeutic approaches. Nutrients.

[37] Stompor T., Sulowicz W., Dembinska-Kiec A., Janda K., Wojcik K., Zdzienicka A. (2003). An association between body mass index and markers of inflammation: Is obesity the proinflammatory state in patients on peritoneal dialysis?. Perit. Dial. Int..

[38] Kalantar-Zadeh K., Kilpatrick R.D., Kuwae N., Wu D.Y. (2005). Reverse epidemiology: A spurious hypothesis or a hardcore reality?. Blood Purif..

